# Immune Modifying Effect of Drug Free Biodegradable Nanoparticles on Disease Course of Experimental Autoimmune Neuritis

**DOI:** 10.3390/pharmaceutics14112410

**Published:** 2022-11-08

**Authors:** Ehsan Elahi, Mohamed Ehab Ali, Julian Zimmermann, Daniel R. Getts, Marcus Müller, Alf Lamprecht

**Affiliations:** 1 Department of Neurology, University Clinic Bonn, Campus Venusberg 1, 53127 Bonn, Germany; 2 Department of Pharmaceutics, Institute of Pharmacy, University of Bonn, Gerhard-Domagk Str. 3, 53121 Bonn, Germany; 3 Myeloid Therapeutics, 300 Technology Sq., Suite 203, Cambridge, MA 02139, USA

**Keywords:** Guillain-Barré Syndrome, experimental autoimmune neuritis, macrophages, poly(lactic-co-glycolic) acid, nanoparticles

## Abstract

Guillain-Barré Syndrome (GBS) is an autoimmune disease of demyelination and inflammation of peripheral nerves. Current treatments are limited to plasma exchange and intravenous immunoglobulins. Cargo-free nanoparticles (NPs) have been evaluated here for their therapeutic benefit on the disease course of experimental autoimmune neuritis (EAN), mimicking the human GBS. NPs prepared from poly-lactic co-glycolic acid (PLGA) with variable size and surface charge (i.e., 500 nm vs. 130 nm, polyvinyl alcohol (PVA) vs. sodium cholate), were intravenously administered in before- or early-onset treatment schedules in a rat EAN model. NP treatment mitigated distinctly the clinical severity of EAN as compared to the P2-peptide control group (P2) in all treatments and reduced the trafficking of inflammatory monocytes at inflammatory loci and diverted them towards the spleen. Therapeutic treatment with NPs reduced the expression of proinflammatory markers (CD68 (P2: 34.8 ± 6.6 vs. NP: 11.9 ± 2.3), IL-1β (P2: 18.3 ± 0.8 vs. NP: 5.8 ± 2.2), TNF-α (P2: 23.5 ± 3.7 vs. NP: 8.3 ± 1.7) and elevated the expression levels of anti-inflammatory markers CD163 (P2: 19.7 ± 3.0 vs. NP: 41.1 ± 6.5; all for NP-PVA of 130 nm; relative to healthy control). These results highlight the therapeutic potential of such cargo-free NPs in treating EAN, which would be easily translatable into clinical use due to their well-known low-toxicity profile.

## 1. Introduction

Guillain-Barré Syndrome (GBS) is an autoimmune inflammatory disorder that causes inflammation and demyelination of the peripheral nervous system (PNS). Although the underlying immunopathogenesis that causes peripheral nerve damage is not yet completely understood, it is known that macrophage and T-cell infiltration is linked with a monophasic demyelination of peripheral nerves [1]. Much like GBS, experimental autoimmune neuritis (EAN) also involves the infiltration of macrophages and T-cells into the peripheral nervous system and breaching of the blood-nerve barrier, resulting in inflammatory demyelination of the peripheral nerves [2].

The critical role of macrophages in the pathogenesis of GBS and EAN has been determined by a plethora of studies. During the early stages of GBS, M1 macrophages promote cellular toxicity and increase the proinflammatory cytokines, resulting in the demyelination of nerves. Whereas, in the later stages of GBS, M2 macrophages are associated with the recovery of disease by promoting the secretion of anti-inflammatory cytokines [2]. Therapeutic options to treat GBS are limited. Plasma exchange and intravenous immunoglobulins (IVIG) are recommended therapies for treatment of GBS. Despite the effectiveness of these treatment options, the course of GBS is long and complicated [3,4]. Therefore, more effective as well as less invasive therapeutic approaches are required to improve the treatment of GBS patients.

Nanoparticles (NPs) with immune-modulating properties are emerging as a potential therapeutic option in immune-mediated diseases. Recent experimental studies which used NPs to modulate the immune-mediated disorders, attained some prominence of attention when drug-free NPs proved their immunomodulatory efficacy by passively targeting various immune disease models such as inflammatory bowel disease, experimental autoimmune encephalomyelitis (EAE), West-Nile virus encephalitis, spinal cord injury models and sodium thioglycolate-induced peritoneal inflammation [5,6,7]. 

Intravenously administered cargo-free NPs bind with circulating monocytes that change the degree of expression of phosphatidylserine on their surface, resulting in a redirection of the monocytes from lesion site to spleen [7]. Macrophages constitute an important part of the immune system by phagocytosing the foreign moieties like NPs. Nevertheless, it is as yet unclear how macrophages of distinct phenotypes such as M1 or M2 interact with NPs with variable physicochemical properties. Strategies to selectively target the subpopulation of macrophages still require further exploration [8]. The uniqueness of this approach is the use of drug-free NPs as compared to the other contemporary particle treatments in which NPs are loaded with specific adjuvants [7]. Using NPs with passive targeting mainly relies on physicochemical properties e.g., charge and size [9]. The biocompatibility and biodegradability of the matrix material is surely a primary necessity for intravenous administration of NPs and accordingly, classical candidates seem to be of first choice, such as poly-lactic co-glycolic acid (PLGA) which has been used in this study [10].

The immunomodulatory potential of drug-free PLGA-NPs is yet unproven in EAN but seems promising based on the success of immune-modifying NP approaches in other diseases [5,7]. Accordingly, we have tested PLGA-NPs in different treatment paradigms, such as before- and early-onset treatment of EAN in order to evaluate potential impacts by particle size, surface charge or surfactants. 

## 2. Materials and Methods

### 2.1. Materials

PLGA (Resomer RG503H was purchased from Evonik AG (Germany); PVA (Mowiol 4-88), ethyl acetate and sodium cholate were purchased from Sigma-Aldrich, Germany and was of analytical grade. P2 peptide was purchased from Institute of Medical Immunology, Charité, Berlin, Germany. CFA (Complete Freund’s adjuvant) was purchased from Sigma Aldrich, Germany. Inactivated Mycobacterium tuberculosis particles dissolved in mineral oil was purchased from Difco Laboratories GmbH, Germany. For histology, primary anti-rat antibodies (CD68, MHC-II (OX-6), CD43 (W3/13)) were purchased from BioRad, Germany. For flow cytometry analysis CD16/CD32, CD45 (OX-1, Pecy5) and CD11b (OX-42, PeCy7) were purchased from BD Bioscience, Germany. CD68 (ED1, PE) was purchased from eBioscience, Germany. CD163 (ED2, PE) was purchased from Bio-Rad, Feldkirchen, Germany). Primers for RT-PCR (CD68, CD163, IL-1β, IFN-γ, IL-17, TNF-α) were purchased from Bio-Rad, Germany).

### 2.2. Preparation and Characterization of NPs

#### 2.2.1. Preparation of NPs

NPs were prepared by the conventional oil-water emulsion solvent evaporation technique (Figure S1) [11]. A quantity of 100 mg PLGA was first dissolved in 5 mL of ethyl acetate, forming the organic phase. This organic solution was then poured into 10 mL of an aqueous surfactant solution, containing either polyvinyl alcohol (PVA) or sodium cholate. A primary coarse emulsion was then further homogenised using ultrasonic cell disruptor (Banoelin sonopuls, Berlin, Germany) for 2 min on an ice bath, followed by solvent evaporation under reduced pressure using a Büchi Rotavapor RE 120 (Büchi, Flawil, Switzerland). The preparation conditions were adjusted to produce NPs with monomodal size distributions and mean diameters of around 130 and 500 nm by changing the concentration of surfactant. This was achieved by using 1% (*w*/*v*) or 0.2% (*w*/*v*) of PVA to obtain NPs with a nominal diameter of 130 and 500 nm, respectively, while sodium cholate was used at concentration of 0.1% *w*/*v* in the aqueous phase. Unbound surfactant was removed from the supernatant by a dialysis step (membrane cut-off: 100 kDa), formulations were then freeze dried without cryoprotectant, and redispersed in isotonic phosphate buffered saline (pH 7.4) prior to injection. Table 1 shows the surfactant, size and zeta potential of each nanoparticle type.

#### 2.2.2. Determination of the Particle Size and Zeta Potential

NPs were analysed for their particle size and size distribution in terms of the average volume diameters and polydispersity index (PI) by photon correlation spectroscopy using particle size analyser (Brookhaven Instruments Corporation, Holtsville, NY, USA) at a fixed angle of 90° at 25 °C. The nanoparticle suspension was diluted with distilled water before particle size analysis. Samples were diluted with a solution containing sodium chloride to adjust the conductivity to 50 μS/cm for zeta potential measurement. All samples were analysed in triplicates at 25 °C and the error was calculated as standard deviation (S.D).

### 2.3. Animals and Ethics

Experiments were carried out using female Lewis rats, 6–8 weeks old. The animals were purchased from Charles River Laboratories (Sulzfeld, Germany). A maximum of four rats were housed in plastic cages. Cages were kept in the room under controlled illumination (light-dark cycle: 12:12 h) and environmental conditions (temperature: 22 ± 2 °C; humidity: 55 ± 5%). Water (ad libitum) and food pellets were freely available for all rats. Animals were housed for at least four days before the start of the experiments.

### 2.4. Disease Induction

#### 2.4.1. Antigen

Immunogenic P2 peptide (amino acids 53–78) (RTESPFKNTEISFKLGQEFEETTADN) (Institute of Medical Immunology, Charité, Berlin, Germany) dissolved in sterile phosphate-buffered saline (PBS) to a concentration of 2 mg/mL was used for P2-peptide immunization.

#### 2.4.2. Immunization with P2-Peptide

P2-peptide (amino acids 53–78) emulsified with an equal volume of CFA (Complete Freund’s adjuvant) (Sigma Aldrich, Germany) containing inactivated Mycobacterium tuberculosis particles (Difco Laboratories GmbH, Germany) in mineral oil was used to enhance the immunogenic effect of P2-peptide immunisation. The resulting emulsion was tested by placing one drop of emulsion on the surface of water at room temperature. The drop of emulsion was not allowed to disperse. If it dispersed on the surface of water, then it would not be stable and hence not suitable for injection. P2 administration into hind paws and tail root were performed under the anaesthesia of Isoflurane (Piramal Critical Care Deutschland GmbH) 3% in 95% oxygen. All efforts were made to minimise the number of animals used and minimise their suffering. In the first two experimental studies, 100 µg of P2-peptide emulsified with CFA containing 1 mg/mL of inactivated mycobacterium tuberculosis particles was injected into each hind paw of the rats. In the following studies 100 µg of P2-peptide emulsified with CFA containing 3 mg/mL mycobacterium tuberculosis particles was injected into each hind paw of the rats and 200 µg was injected into the tail root under the anaesthesia.

### 2.5. NPs Administration and In-Vivo Study Layout

After immunization with P2-peptide, PLGA-NPs were resuspended in PBS to a dose of 9 mg/kg (which was calculated by using an equation of body surface area [12] with a final concentration of 6.2 mg/mL and administered to animals into the tail vein root which reflected the NP concentration after resuspension of NP after freeze-drying by considering the rat weight. We have conducted five in vivo experimental studies with different treatment goals. At first, we determined the therapeutic efficacy of 9 mg/kg NP-PVA500 in the period before onset and in the early-onset treatment of EAN. Then, in the third experiment, we used two different sizes (NP-PVA500, NP-PVA130) of NPs (9 mg/kg) to determine their efficacy in the early-onset treatment of EAN. While in the fourth experiment, we opted for 9 mg/kg of NP-PVA130 to treat the animals in the before-onset treatment of EAN. Then, in the fifth experiment, we used NPs bearing different surfactants (NP-PVA130, NP-Chol130) in the treatment of EAN. In each experiment, we divided the animals into three main groups: one was the control group, which did not receive any treatment, another was the NPs treatment group in which P2-peptide immunized animals were treated with immune modifying PLGA-NPs, while the last was the P2-peptide group, in which animals only received P2-peptide immunization and did not receive any particle treatment. All the rats were monitored carefully for any unwanted effects until the end of the experiment.

### 2.6. Clinical Score Assessment

Animals were weighed and scored for disease severity daily from the day of immunisation till the end of the experiment. Disease progress and propensity was assessed clinically employing a scale ranging from zero to 10 originally described by Enders [13]: 0, normal; 1, hanging tail-tip; 2, tail paralysis; 3, absent righting/inability to sit up; 4, gait ataxia abnormal position; 5, mild paraparesis; 6, moderate paraparesis; 7, severe paraplegias; 8, tetra paresis/complete paralysis of all extremities; 9, moribund; 10, death.

### 2.7. Histological Analysis

#### 2.7.1. Tissue Processing for Histology

The tissues for histological assessment, such as routine histology and immunohistochemistry, were collected from each group of animals (P2-group, NP-group, and control). Immediately afterwards, euthanasia sciatic nerves were collected from animals, fixed overnight in PBS buffered 4% paraformaldehyde at 4 °C, and were later washed with PBS. For H&E (Haematoxylin and eosin) and immunohistochemistry on cryosections, tissues were embedded with Tissue Tek^®^ (Sakura Finetek, Staufen, Germany) and snapped frozen in liquid nitrogen. Sections (10µm) were prepared for H&E and immunohistochemical analysis by using Leica microtome CM3050 S (Leica Biosystems, Germany).

#### 2.7.2. Routine Histology and Immunohistochemistry

Frozen nerve sections were stained with H&E for routine histological assessments. For immunohistochemistry, cryosections were first fixed with ice-chilled methanol/acetone. Endogenous peroxide was inhibited by 0.5% H_2_O_2_ in methanol (Merck, Darmstadt) for 10 min. Sections were incubated with 10% bovine serum albumin to block nonspecific binding of immunoglobulins for 30 min at room temperature, followed by overnight incubation with primary antibodies (1/100) at 4 °C. Antibodies binding to tissue sections were visualised with secondary biotinylated antibodies (donkey anti-mouse; 1/200). Sections were treated with a horseradish peroxidase-conjugated Streptavidin complex (Vector Laboratories) (1/200) for 45 min. Signals were visualised by NovaRED colouring reagent (Axxora, Lörrach, Germany) according to the manufacturer’s instructions. Conventional and immunohistochemical-stained sections were examined under a bright field and fluorescent microscope, E-800 Nikon eclipse (Nikon, Dusseldorf, Germany). Bright-field images were acquired using a 15.2 SPOTFLEX camera. The acquired fluorescence signals were merged using SPOT advanced 4.5 software (Diagnostic Instruments, Sterling, MI, USA).

### 2.8. Flow Cytometric Analysis of Blood and Splenic Tissue

To determine the percentage of co-localization of NPs into the cells, characterization of the immune cell population and the paradigm shift of macrophages, we used fluorescence-activated cell sorting (FACS). At the end of the period before the onset and the early-onset treatment experiment (day 17 post immunisation) with both the sizes of NPs (PVA500, PVA130), rats were anaesthetised, and blood was drawn intracardially, which was collected into tubes containing EDTA (Ethylenediaminetetraacetic acid). EDTA tubes containing blood samples were centrifuged at 500× *g* at 4 °C and the supernatant was discarded. Then the samples were re-suspended into 1 mL of 1X PBS buffer solution and transferred to FACS tubes. Approximately 2mL of RBC lysis buffer (BD Bioscience, San Jose, USA) with ratio of 1:10 (dist. Water) was added and incubated at room temperature for 10 min. Then the FACS buffer was added, and samples were subjected to centrifugation at 800× *g* for 10 min at 4 °C. 100 µL of sample was collected and blocked with CD16/CD32 (Fc block; BD Bioscience) antibody. Staining antibodies were added with a ratio of 1:100. Samples were incubated with fluorochrome-conjugated antibodies to detect CD45 (OX-1, Pecy5) and CD11b (OX-42, PeCy7) (BD Bioscience). NPs were conjugated with FITC dye and therefore, separated in the FITC channel. After staining, antibody bound samples were detected using a BD FACSCanto II (BD Bioscience), and the gathered data was analysed using the flow cytometry software FlowJo 10 (TreeStar, San Carlos, CA, USA).

In the flow cytometric analysis of splenocytes, animals were euthanized under anaesthesia (3% isoflurane, 95% oxygen) and spleens were removed carefully and placed in an ice-chilled petri dish containing 1XHBSS (Hank’s Balanced Salt Solution; Gibco Life Technologies, Darmstadt, Germany). Spleen tissue was cut into pieces and homogenised using a needle (0.6 × 25) and a syringe (5 mL) before passing it through a 70µm cell strainer (BD Bioscience, Heidelberg, Germany). The RBC lysis buffer was added into room temperature incubated splenocytes and then washed with PBS at 500 g for 10 min at 4 °C. Supernatant was discarded, and 100 µL of the sample was collected from each sample tube and blocked with a 1:100 ratio of CD16/CD32 (Fc block; BD Bioscience) antibody. Staining antibodies were added with a ratio of 1:50. Samples were incubated with fluorochrome-conjugated antibodies to detect CD45 (OX-1, Pecy5), CD11b (OX-42, PeCy7).

To determine the co-localization of NPs with surfactant, at the end of early-onset treatment (day 17 post immunisation) with NPs with different surfactant (NP-130PVA, NP-Chol130), we performed flow cytometric analysis of splenocytes by following the above-mentioned protocol. Supernatant was discarded, and 100 µL of the sample was collected from each sample tube and blocked with a 1:100 ratio of CD16/CD32 (Fc block; BD Bioscience) antibody. Staining antibodies were added with a ratio of 1:50. Samples were incubated with fluorochrome-conjugated antibodies to detect CD45 (OX-1, Pecy5), CD11b (OX-42, PeCy7). (BD Bioscience), CD68 (ED1, PE) (eBioscience) CD163 (ED2, PE) (Bio-Rad AbD Serotec GmbH, Puchheim, Germany). PLGA-NPs were conjugated with FITC dye, and therefore, separated in the FITC channel. After staining, antibody-bound samples were detected using a BD FACS Canto II (BD Bioscience), and gathered data was analysed using the flow cytometry software FlowJo 10 (TreeStar, San Carlos, CA, USA).

### 2.9. Determination of Inflammatory Markers and Cytokines by Real Time-PCR

Sciatic nerves from all animal groups (Control, P2, NP-PVA-130) were collected and snapped frozen in liquid nitrogen. Total RNA was isolated from the whole sciatic nerve using Trizol (Sigma-Aldrich) reagent method. Total RNA (1 µg) was reverse transcribed into cDNA by using SuperscriptTM III reverse transcriptase (Invitrogen, Germany). Quantitative PCR assays were carried out by using SYBRgreen. The composition of reaction mixture contained 1.5 µL of cDNA, 1 µL of each primer, 7.5µL Distilled H_2_O, 10 µL of 2X SYBR Green PCR SsoAdvanced Universal SYBR Green Supermix (Applied Biosystems, Darmstadt, Germany) in a total volume of 20 µL. For Rt-PCR assay a pre incubation period of 2 min at 95 °C was chosen and 39 PCR cycles (5 min at 95 °C, and 30 min at 60 °C) were performed at CFX connect Real-Time PCR detection system (Applied Biosystems, Darmstadt, Germany). In each cycle, the SYBR Green fluorescence signal was measured. Samples were analysed simultaneously for GAPDH mRNA as housekeeping internal control. The mRNA levels for each target were normalized to mRNA levels of GAPDH and expressed relative to that of control animals. Each sample was assayed in duplicate. Data was determined as the mRNA fold of change ± SEM.

### 2.10. Statistical Analysis

We performed various statistical analyses and generated several graphs for our data using GraphPad Prism (GraphPad Software, San Diego, CA, USA). For statistical analysis of clinical scoring data, we conducted a Mann-Whitney nonparametric *U*-test while for RT-PCR, a Student’s *t*-test (unpaired) was used. For the FACS data analysis, we performed a one-way ANOVA with a Bonferroni post-test and *t*-test (unpaired). Different levels of significance are given in the respective result sections, *p* ≤ 0.05 (*), *p* ≤ 0.01 (**) or *p* ≤ 0.005 (***).

## 3. Results

### 3.1. Mild Disease Scenario

#### 3.1.1. Before-Onset Treatment with NP-PVA500

**Figure 1:**
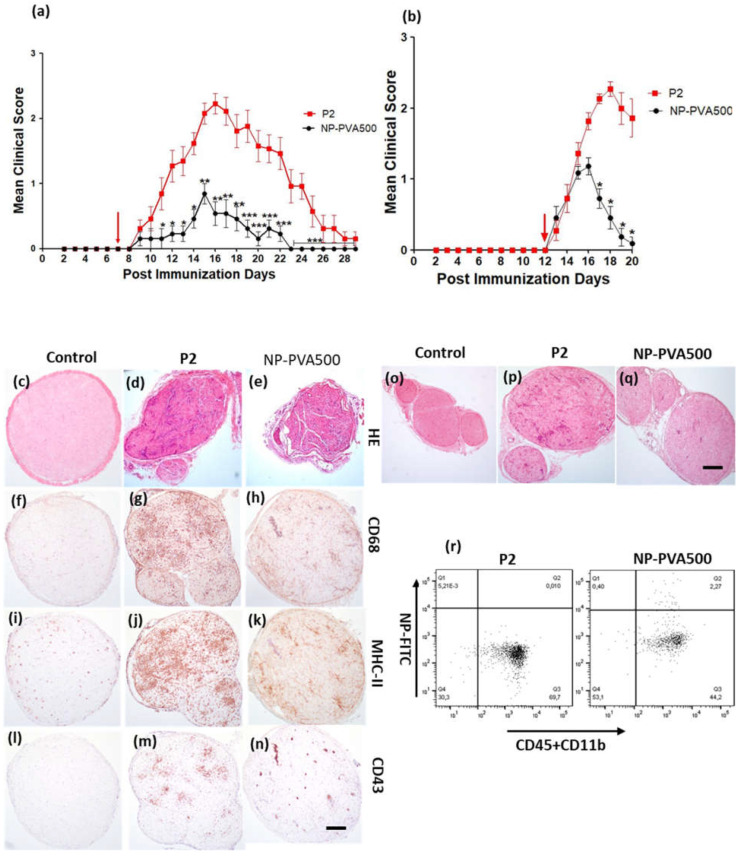
Treatment with NP-PVA500 before and at early onset of EAN symptoms. (**a**) Mean clinical score (± S.E.M.) post-immunization with P2-peptide and NPs. The P2-peptide (*n* = 13) group was administered with P2-peptide only. The NP-PVA-500 (*n* = 13) group, in addition with P2-peptide, was administered with a daily intravenous 9 mg/kg dose of NP-PVA500 before the onset of EAN. (**d**,**e**) Histological changes in the sciatic nerves of P2-peptide and NP-PVA-500 treated animals and co-localization of infiltrates with routine H&E histology A high titer of activated macrophages (CD68) was observed in the P2-peptide group (**g**) in contrast to a smaller proportion of macrophages found in the NP-PVA500 group (**h**), while in the control animals (**f**) no such response was observed. A clear difference in MHC-II (OX-6) positive cell population between the P2-peptide group and the -NP-PVA500 group was observed (**j**,**k**). A considerable accumulation of T-cells (CD43+) was observed in P2 animals (**m**) at the end of the experimental phase, while a lesser population of T-cells was observed in the NP-PVA500 group (**n**) at the same time. Treatment with NP-PVA500 at the early onset of EAN: (**b**) Mean clinical scores (± S.E.M.) of P2-peptide (*n* = 11) group and NP-PVA500 treated group (*n* = 11). Early-onset treatment with a 9 mg/kg dose of NP-PVA500reduced the perivascular infiltration of mononuclear cells (**q**). A high titer of accumulation of infiltrating monocytes across the perivascular region of the sciatic nerves was observed in P2-Peptide treated rats (**p**). The control group didn’t exhibit any histological changes (**c**,**f**,**i**,**l**,**o**). (The arrow indicates the start of NPs treatments.) (**r**) Dot plots of the flow cytometric representation of the percentage of the extent of NP-FITC+ monocytes (blood). Magnification 10x. Scale bar represents 50μm. Mann-Whitney nonparametric *U*-test. * *p* ≤ 0.05, ** *p* ≤ 0.01, *** *p* ≤ 0.005.

The “before-onset” treatment of EAN was started at the seventh day of post-immunization with a 9 mg/kg dose, while early EAN symptoms appeared at around days nine to eleven. Treatment with NP-PVA500 was continued until the end of the experiment. Even though a mild disease severity was observed, there was a significant difference in the mean clinical scores of the NP-PVA500 treatment group as compared to the P2-peptide group. The peak clinical score was 2.46 ± 0.14 in the P2 group and 1.30 ± 0.13 in the NPs treated group (Figure 1a).

Histologically, a high level of infiltration of activated macrophages (CD68) was observed in the perivascular region of the sciatic nerves of P2-peptide group animals as compared to NP treated animals (Figure 1g,h). A substantial accumulation of CD43 and MHC-II positive cell population was observed in the sciatic nerves of the P2-peptide group (Figure 1j,n). In contrast, a low-grade accumulation of CD43 and MHC-II cells was observed in the sciatic nerves of the NP-PVA500-treated animals (Figure 1n,k). In the treatment with NP-PVA500, a minor extent of infiltration of inflammatory cells was observed (Figure 1e) as compared to the P2-peptide group where an excessive accumulation of mononuclear cells in the perivascular region of the sciatic nerve was observed. (Figure 1d). The control group of both the treatments did not exhibit any histological changes (Figure 1c,f,i,l). Besides, we also observed that NP-PVA500 were internalized by the circulating monocytes (CD45+CD11b+) (Figure 1r).

#### 3.1.2. Early-Onset Treatment with NP-PVA500

In the “early-onset” treatment experiment, treatment was started at day 12 when the first clinical symptoms of EAN occurred. The overall disease severity was milder in the P2-peptide group, achieving a maximal mean clinical score of 2.40 ± 0.13, whereas in the NP-PVA500 treated animals, the mean peak clinical score was 1.18 ± 0.12 (Figure 1b). An early decline phase was observed with NP treatment. A low accretion of inflammatory cells was observed in the NP-PVA500 treatment group (Figure 1q) as compared to the P2-peptide group (Figure 1p). The sciatic nerves of the control group did not show any inflammatory lesions or histological changes in their nerve milieu (Figure 1o).

### 3.2. Severe Disease Scenario 

#### 3.2.1. Early-Onset Treatment with Different Sizes (NP-PVA500, NP-PVA130)

**Figure 2:**
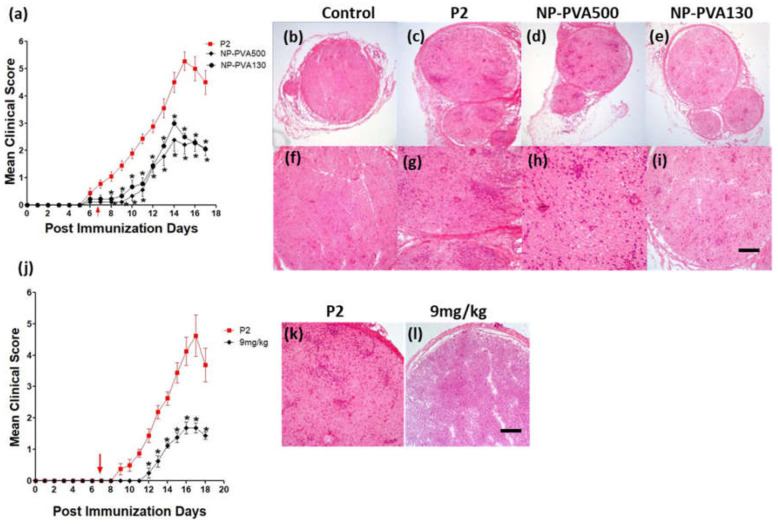
Treatment with different sizes of NPs at the early onset of EAN. (**a**) Mean clinical scores (± S.E.M.) post-immunization with P2-peptide (*n* = 9) and treatment with different sizes of NPs (*n* = 9). An intravenous daily 9 mg/kg dose of both sizes of NPs, NP-PVA500 and NP-PVA130, were administered to treatment group animals at the onset of the disease. (**d**,**h**) Early-onset treatment with NP-PVA500500 diminished the inflammatory response in sciatic nerves of treated rats. (**e**,**i**) Treatment with NP-PVA130also lowered the extent of inflammatory response in sciatic nerves of treated rats. Before-onset treatment with 9 m/kg of PVA130. (**j**) Mean clinical scores (± S.E.M.) post-immunization with P2-peptide (*n* = 8) and treatment with 9 mg/kg of NP-PVA130 (*n* = 8). Daily infusion of NPs was administered before the onset of disease. Before-onset treatment mitigated the disease severity and reduced the perivascular infiltration of inflammatory cells across the perivascular region of NP-treated sciatic nerves as compared to P2-peptide group (**l**). A clear inflammatory response and accumulation of infiltrates have been observed in sections of the P2-Peptide group (**c**,**g**,**k**). The control group (**b**,**f**) didn’t exhibit any histological changes. (Arrow indicates the start of NPs treatments.) Magnification 10× (**b**–**e**), 20×(**f**–**i**,**k**,**l**). Scale bar represents 50 μm. Mann-Whitney nonparametric *U*-test, * *p* ≤ 0.05.

We observed a low clinical severity of EAN disease course when we applied two different sizes of drug-free NPs (NP-PVA500, NP-PVA130) at the early onset of symptoms. Early neurological symptoms of EAN appeared between 7–9 days of post immunisation. Early-onset treatment of EAN with both NPs (500PVA, 130PVA) significantly reduced the peak clinical score as compared to the P2-peptide group (Figure 2a). Peak clinical scores of 5.50 ± 0.33 were observed in the P2 group, whereas peak clinical scores of 2.55 ± 0.38 and 3.00 ± 0.11 were observed in the NP-PVA500 and NP-PVA130 respectively. An early recovery of the disease was observed as a result of treatment with NPs of both sizes. 

A low grade of infiltrates across the perivascular area of the sciatic nerve was observed with the treatment of NP-PVA500 and NP-PVA130 (Figure 2d,e,h,i) as compared to the P2-peptide group (Figure 2c–g). As expected, the control group animals didn’t exhibit any histopathological changes in routine histology (Figure 2b–f).

#### 3.2.2. Before-Onset Treatment with NP-PVA130

Before-onset treatment with NP-PVA130 was started on day 7. Neurological symptoms of EAN appeared between 9–10 days. Before-onset treatment with a 9 mg/kg dose of NP-PVA130 significantly reduced the clinical severity of EAN (Figure 2j). Peak score of the P2-peptide group was 4.93 ± 0.58, whereas peak clinical score of NP-PVA130 (9 mg/kg) was 1.75 ± 0.16. A lower grade of inflammatory response was observed in before-onset treatment with 9 mg/kg dose of NP-PVA130 (Figure 2l) as compared to the P2-peptide group (Figure 2k).

#### 3.2.3. Early-Onset Treatment Effect with Surfactant Modified NPs on EAN and Their Impact on the Percentage Expression of Pro- and Anti-Inflammatory Macrophages

**Figure 3:**
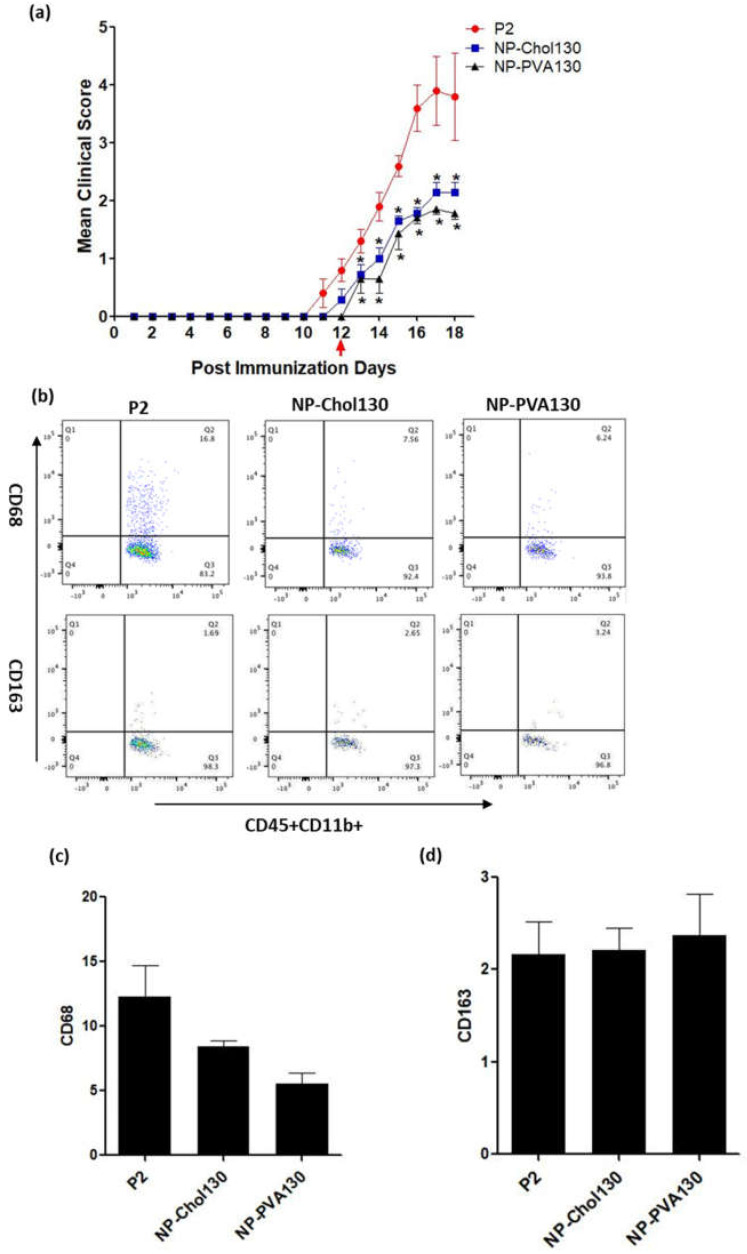
Surfactant modification improved the disease course of EAN. (**a**) Mean clinical scores (±S.E.M.) post-immunization with P2-peptide and treatment with surfactant modified NPs. Symptoms of experimental autoimmune neuritis (EAN) appeared between 10–12 days of post-immunization in each group P2 (*n* = 5), NP-Chol130 (*n* = 7), NP-PVA130 (*n* = 7). Each group was administered with a 9 mg/kg dose of NP-Chol130 and NP-PVA130 at the early onset of disease. Arrow indicates the start of NPs treatment. Dot plots of flow cytometric representation of CD68 and CD163 percentages in CD11b population (**b**) Spleen mononuclear cells (MNCs) were isolated from the P2-Peptide and treatment groups at day 17 of post-immunizations. With flow cytometry, these monocytes were identified by gating against the side and forward scatter. The percentage (Q2) of CD68 and CD163 of each NPs type was determined and compared with the P2-peptide group. A distinct reduced percentage of activated macrophages (CD11b+CD68+) was observed with the treatment of NP-PVA130. A slightly higher CD11b+CD163 cell percentage was observed with the treatment of NP-PVA130. No significant differences were observed between the respective groups. Flow cytometry data represents the percentage expression of activated macrophages (**c**), (CD11b+CD68+) (**d**), CD11b+CD163 of both NP types and P2-peptide treated group comprising of three female Lewis rats per group. For clinical scores Mann-Whitney nonparametric *U*-test, for FACS one-way ANOVA with a Bonferroni post-test, * *p* ≤ 0.05.

Since the NPs bearing Polyvinyl alcohol (PVA) as surfactant yielded promising results, we proposed an experimental study to evaluate the therapeutic efficacy of 130 nm NPs bearing different surfactants (PVA, sodium cholate). Early symptoms of EAN appeared between 11–12 days post immunization. A dose of 9 mg/kg with different NPs was administered at disease onset. A significant improvement in the clinical manifestation of EAN was observed in this experiment (Figure 3a). Peak clinical score of P2-peptide was 4.40 ± 0.67, whereas the peak clinical score of NP-Chol130 was 2.14 ± 0.17 and NP-PVA130 was 1.92 ± 0.07.

To determine and quantify the relative changes in the percentage distribution of specific immune cell populations, spleens harvested at peak disease (day 17 post immunization) level were analysed for a paradigm shift in the monocyte proliferation. We observed a considerable reduction in the pool of activated macrophages (CD11b+CD68+) in the spleens of nanoparticle treatment groups as compared to the P2-peptide group (12.32 ± 2.38 *n* = 3) (Figure 3c). Reduction in percentage distribution of activated macrophages was observed with NP-PVA130 (5.53 ± 0.87 *n* = 3) and NP-Chol130 (8.40 ± 0.47 *n* = 3) NPs. We also observed a slight increase in the percentage of the expression of CD11b+CD163 cells in NPs treatment groups, NP-PVA130 (2.37 ± 0.44 *n* = 3) and NP-Chol130 (2.17 ± 0.29 *n* = 3) as compared to the P2 peptide group (1.78 ± 0.35 *n* = 3) (Figure 3d).

### 3.3. Determination of the Percentage of Co-Localization of NPs into the Immune Cells

**Figure 4:**
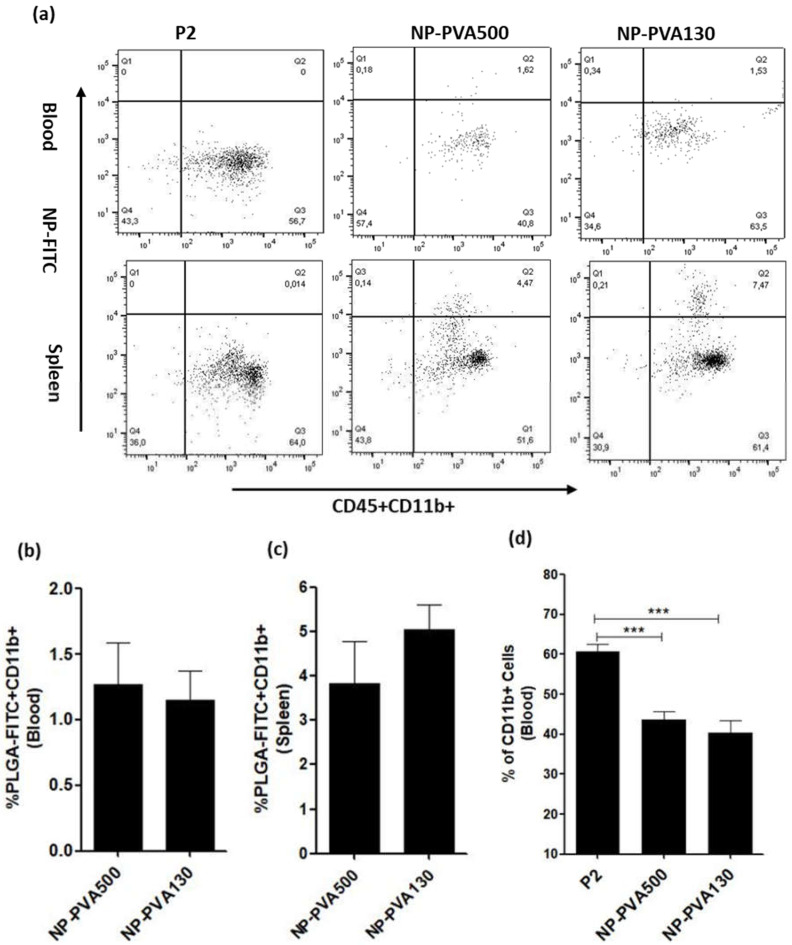
Dot plots of the flow cytometric representation of the percentage of the extent of NP-FITC+ monocytes: (**a**) Blood and spleen mononuclear cells (MNCs) were isolated from the P2-peptide and different sized NPs (NP-PVA500, NP-PVA130). For the treatment groups at day 17 of post immunizations, the percentage (Q2) of PLGA-FITC+CD11b+ cells of each group was determined. The data of the six animals is presented in the figure above. (**b**,**d**) Data of the percentage of the extent of NP-FITC+ monocytes of both in blood and spleen; (**b**,**c**) *t*-test (unpaired); (**d**) one-way ANOVA with a Bonferroni post-test, *** *p* ≤ 0.005.

Since both the sizes of the NPs reduced the disease severity and perivascular infiltration of inflammatory cells, we determined the percentage of the extent of co-localization of NPs into the immune cells in the blood and spleen. We observed that NPs were internalised by the circulating monocytes (CD45+CD11b+) (Figure 1r) and were diverted to the spleen, where a higher percentage of these monocytes was observed (Figure 4a). Whereas P2-peptide treated animals did not receive the nanoparticle regimens and didn’t show any particle signal (Figure 4a). Moreover, when we compared the percentage of the extent of co-localization of both sizes i.e., NP-PVA500 (1.27 ± 0.31 *n* = 6) and NP-PVA130 (1.15 ± 0.22 *n* = 6) in the blood (Figure 4b), we observed no significant difference. Whereas a higher percentage of PLGA-FITC+CD11b+ monocytes was observed in the spleens of both the sizes of NPs i.e., NP-PVA500 (3.84 ± 0.93) and NP-PVA130 (5.04 ± 0.56) (Figure 4c). Apart from the internalization of NPs into immune cells, we also observed a significantly lower percentage of CD11b+ monocytes in the blood samples of NPs (PVA500, PVA130) treated animals as compared to P2-peptide group where a higher percentage of circulating CD11b+ monocytes was observed (Figure 4d).

### 3.4. Treatment with NP-PVA130 Modulates the Local Immune Response in EAN

**Figure 5:**
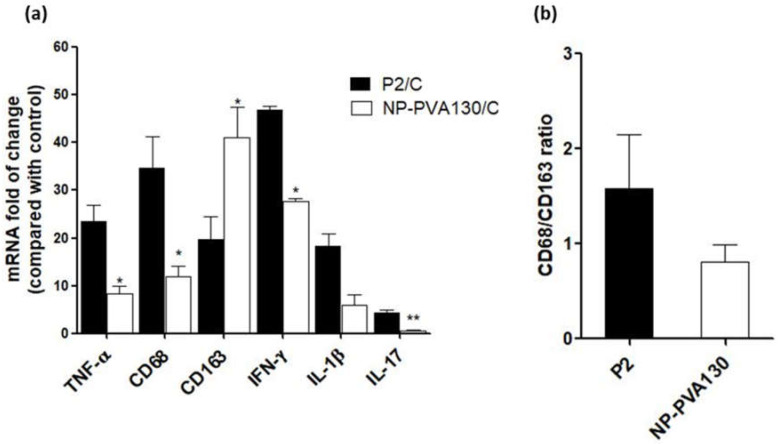
(**a**) Treatment with NP-PVA-130 attenuated the expression levels of pro-inflammatory markers and increased the expression levels of anti-inflammatory markers. (**b**) mRNA expression ratio of CD68/CD163. (*n* = 3), *t*-test (unpaired) * *p* ≤ 0.05, ** *p* ≤ 0.01.

We conducted a quantitative real-time PCR assay to compare the RNA expression levels of the selected pro- and anti-inflammatory markers in the sciatic nerves of PLGA-NP-PVA130 (*n* = 3) and P2-peptide treated rats (*n* = 3). Expression levels (fold increases of mRNA expression) of each respective marker from both the particle treatment and P2-peptide group were normalized with the below-mentioned specific markers’ expression levels in the control group (Figure 5a). The expression level of selected markers is listed below. We then calculated and compared the mRNA expression ratio of CD68 versus CD163 in the P2-peptide treated animals and the NP-PVA130 treated animals. CD68/CD163 ratio comparison showed a higher expression of M1 phenotype macrophages in the P2-peptide group, whereas a smaller expression of M1 macrophages was observed in the NP-PVA130 treatment group (Figure 5b).

## 4. Discussion

GBS is an acute paralytic autoimmune disease that mainly affects the axons and myelin, predominantly causing demyelination and axonal nerve damage in the PNS. GBS represents a variety of subtypes depending on involvement of nerve fiber. These subtypes vary in their clinical, histological and electrophysiological aspects [14]. EAN is an immunological animal model which resembles many clinical, immunological, and histological as well as electrophysiological aspects of most occurring subtypes of GBS, that is, acute inflammatory demyelinating polyradiculoneuropathy (AIDP) [15]. In this in vivo study, NPs varying in treatment approaches, i.e., before- and early-onset treatment, size, and surfactant ameliorated the severity of EAN.

At first, we examined the therapeutic potential of NP-PVA500 when applied before onset and at early onset of EAN. Though the disease severity was milder, a significant difference in the peak clinical score was observed with the NPs as compared to the P2-peptide group. Before-onset treatment with NP-PVA500 suppressed the infiltration of T cells, macrophages (CD68+) and reduced the levels of MHC-II into the sciatic nerve. The endoneurial accumulation of T cells and macrophages is essential for the development of EAN [16,17]. Comparable to other findings [5], we suggest that the reduction of the accumulation of inflammatory cells into the sciatic nerves of the NPs treated animals might be due to the ‘mopping-effect’ mechanism of NPs, where their adsorption to circulating monocytes can reduce their presence at inflammatory loci leading to amelioration of disease. During EAN, MHC-II antigens are highly upregulated on macrophages [18]. We observed a lesser extent of MHC-II (OX-6) levels in NP-PVA500 treated animals than in P2 treated animals. Restriction in inflammatory monocyte trafficking towards the perivascular area was also observed with our before- and early-onset treatment with NP-PVA500.

We observed that early-onset treatment with both sizes of NPs reduced the clinical course of EAN and less histological damage was observed. Moreover, both sizes of NPs were internalized by circulating monocytes (CD45+CD11b+). Our findings indicate that the use of different-sized nanoparticles does not show a significant difference in the immune-modulating effect of NPs. Before-onset treatment with NP-PVA130 also mitigated the disease severity and reduced the inflammatory monocytes accumulation across the perivascular region of peripheral nerves. Moreover, reduction in pro-inflammatory response has been reported with the use of negatively charged NPs [19]. It is speculated that their immune-modulating effect may not primarily depend on their size but the surface charge. 

Owing to the numerous surfactant options available to design nanoparticles which can potentially change the treatment outcome, the surface properties of nanoparticles are of prime importance [20]. From our data we observed that our previously used NP-PVA130 and NP-Chol130 not only reduced the clinical course of EAN but also altered the expression of proinflammatory macrophages (M1) more towards the anti-inflammatory (M2) side.

We observed that NPs were internalized by circulating monocytes (CD45+CD11b+) and were diverted to the spleen where a higher percentage of these monocytes was observed. As observed, internalization of these NPs into circulating monocytes diverted them towards the spleen rather than reaching the inflammation loci. Furthermore, a lesser percentage of circulating monocytes in the blood samples of NP-treated animals (NP-PVA500, NP-PVA130) was observed.

Upon intravenous administration, NPs immediately interact with immune cells and plasma proteins (opsonin). Immune cells can uptake NPs through various endocytic pathways and adsorption of plasma proteins to the surface of NPs can facilitate this process [21]. For the selective adsorption of a particular protein the electrostatic interaction between protein and adsorbent can be changed by changing the surface charge of particles. It is known that negatively charged nanoparticles bind with positive sites on the surface of macrophages, recognized by macrophage receptors [22] followed by internalization by monocytes-derived macrophages predominantly through dynamin and clathrin mediated endocytic pathways [23]. Electrostatic interactions between negatively charged nanoparticles and positive sites on macrophages have been considered to be essential for the internalization of particles [5,22].

However, non-opsonic receptors such as scavenger receptors, which are capable of interacting directly with the NP surface moieties, are also expressed on monocytes [23]. Studies have reported that anionic NPs can also be internalized through a non-opsonic receptor present on monocytes i.e., MARCO (macrophage receptor with collagenous structure) receptor and modulate the inflammatory response. It is important to consider that NPs are adsorbed and internalized but are not restricted to caveolae-clathrin mediated endocytosis, micropinocytosis and phagocytosis [7]. It can be also concluded that negatively charged NPs might have selectively targeted the positive receptor site on the surface of circulating monocytes and diverting them to the spleen, resulting in reduction of inflammatory response in PNS which is evident by a low clinical score as well. 

Other experimental therapies aiming at depleting monocytes from the bloodstream were described in the past. A liposomal preparation containing dichloromethylene diphosphonate (Cl2MDP) has been found to be effective in the selective elimination of macrophages and prevented the development of EAN [24]. Consistent with their observations, we also showed that early-onset treatment with both the NPs (PVA500, PVA130) significantly mitigated the severity of EAN and elimination of monocytes from the bloodstream has also been observed with our particle treatment.

A low disease manifestation and expression of CD68 (here considered as an indicator for M1) splenic macrophages was observed with NP-PVA130 and NP-Chol130 as compared to the P2-peptide control. Moreover, we observed a higher expression of CD163 in splenic macrophages with treatment of NP-PVA130 and NP-Chol130 as compared to the P2-peptide control, which in turn argues for M2. From our data, it is reasonable to speculate that NPs exhibited their immune-modifying ability by interfering with the polarization state of the splenic macrophages. However, the exact mechanism of nanoparticle treatment on the state of macrophage polarization needs further exploration.

A considerably smaller disease manifestation and reduction in M1 macrophages was observed with NP-PVA130 as compared to other types of nanoparticles. Interestingly, this observation is in accordance with our RT-PCR data from the sciatic nerves of NP-PVA130 treated animals, where we observed less expression of inflammatory macrophages M1 (CD68) as compared to P2-peptide group. Moreover, a slight shift towards the M2 macrophages was observed in the splenic macrophages. This is also in agreement with our RT-PCR data where we observed a higher expression of M2 (CD163) macrophages in NP-PVA130 treatment as compared to the P2-peptide group. Our findings can be correlated with an EAN treatment study, where a similar splenic macrophage polarization effect has been observed at the peak of disease where the early-onset treatment with dimethyl fumarate (DMF) showed that splenic macrophages were more polarized towards the M2 type [25]. From our data we observed only a minor effect by the change of particle size and charge. In opposition to that, other experimental studies on the EAE model revealed that the charge on the nanoparticle surface plays an important role and induces the amelioration of disease. In their studies, they used positive, neutral, and negatively charged drug free PLGA-NPs (500 nm) in the treatment of EAE in the mice model. From their results, particles with a negative surface charge reduced the severity of the disease and improved the survival rate of animals as compared to other particle treatment groups [5]. This is moreover in line with reported efficacy of negatively charged NPs in other proinflammatory models such as inflammatory bowel disease, West-Nile virus encephalitis, spinal cord injury models and sodium thioglycollate-induced peritoneal inflammation. The reasons for these differences can be manifold, however particle surface charges that typically characterized by a standardized method (like measurements of the zeta potential) reflect in-vivo conditions only partially. Accordingly, effects immunomodulation or biodistribution related to corona formation on the particle surface may modify the behaviour in-vivo, while this depends on a large diversity of factors such as charge, hydrophilicity, material of particle matrix or even the preparation method. However, this requires further exploration. Accordingly, from this study one can only claim a general benefit for the EAN treatment by using cargo-free PLGA-based NPs while the impact of particle properties is minor.

We also observed that early-onset treatment with NPs switched the M1/M2 balance more towards the M2. It has been reported that, macrophage polarisation shifted from M1 to more towards M2 with the use of anionic NPs made up of biodegradable polymers [26]. From our study, we can argue that early-onset treatment with NPs might have suppressed the M1 signalling pathways and upregulated the M2 pathways, due to which we observed an increased expression ratio of M2 macrophages. 

There is a proportional relation between the release of proinflammatory cytokines such as IL-1β, IL-17, IFN-γ and TNF-α and their disease-promoting role in progression of EAN [27]. In agreement with the above notion, we observed that the early-onset treatment with NP-PVA130 also lowered the expression levels of proinflammatory markers in sciatic nerves of the treated animals. It can be speculated that the immune-modifying ability of NP-PVA130 which interferes with the pathological mechanism of EAN is due to the inhibition of inflammatory cytokines or a non-specific adsorptive interaction with them. Keeping this in consideration, we propose that drug-free negatively charged NPs may also be harnessed in the treatment of other inflammatory disorders where the lowering of inflammatory cytokines profile is aimed. However, it can be argued that to bring this therapeutic approach from bench to clinical settings, the use of NP-PVA130 could be an optimal choice due to their smaller size, which is favourable for sterile filtration. It has been reported that 98–100% PLGA nanoparticles with a diameter range of 103–163 nm passed through 0.2 μm membrane filters without having any change in their physical properties such as particle size or distribution [28].

IVIGs are recommended therapies for treatment of GBS. Although the exact mechanism of action of IVIGs is not yet elucidated, IVIG therapy is extensively used in the treatment of GBS patients [4]. Our clinical and histological findings are in agreement with before- and early-onset treatment studies with IVIGs on EAN rat model [29,30]. Although their scoring is different, the clinical score in our before-onset treatment decreased and returned to almost zero on day 22 of NP-PVA500 treated animals, whereas with their before-onset treatment with IVIG the overall clinical score almost returned to zero at around day 25 (Kajii et al., 2014). Similarly, we observed an early recovery phase in our early-onset treatment as compared to their treatment regimen. Methodological differences may account for any minor differences in the clinical outcomes. Although, our clinical scoring method is different as compared to their clinical grading scale and they administered only two infusions (before-onset: 1 mg/kg, early-onset 100 mg/100 gm/body weight of rats) of IVIGs whereas we administered a daily dose of 9 mg/kg of NPs until the end of the experiment, we still believe that the early recovery phase in before- and early-onset treatment could be an encouraging factor in order to translate these PLGA-NPs from bench to clinical settings. Although one should also keep in mind that animal models do not fully reflect the pathophysiology of the human disease, if however, these NPs can ameliorate the disease severity of EAN model and switch the proinflammatory balance more towards anti-inflammatory side then it might a good suggestion to evaluate these NPs in GBS, especially since the therapeutic effect is based on a relatively non-specific mechanism of action. This study provides the first *proof-of-concept* approach for the application of drug-free PLGA nanoparticles in the management of experimental autoimmune neuritis.

## 5. Conclusions

Our study investigated the use of drug free PLGA-based NPs to target and modulate the inflammatory cells to reduce the disease severity of EAN, a GBS model. In this in vivo study, infusions of NPs varying in treatment approaches i.e., before- and early-onset treatment, size, and surfactant but similar in charge (negative) ameliorated the severity of EAN. Results from our study suggested that NPs can be tuned that they can efficiently modulate the circulating monocytes, which is critical to halt the initial inflammatory response. Interestingly, these PLGA based nanoparticles do not need an active pharmaceutical drug. Our study highlighted the importance of cargo-free NPs towards a safe, specific and cost-effective treatment approach to modulate the inflammatory monocytes mediated pathology in various inflammatory disorders and also provided a first hint in view of a potential modulatory role on M1/M2 balance.

## Figures and Tables

**Table 1:** Types of NPs used in our study.

NPs	Surfactant	Size (nm)	Zeta Potential (mV)
NP-PVA500	Polyvinyl alcohol (PVA)	496 ± 12	−33 ± 1
NP-PVA130	Polyvinyl alcohol (PVA)	130 ± 10	−44 ± 1
NP-Chol130	Sodium cholate	132 ± 3	−40 ± 5

## Data Availability

All data are contained within this article.

## Supplementary Materials

The following supporting information can be downloaded at: www.mdpi.com/article/10.3390/pharmaceutics14112410/s1, Figure S1: Schematic presentation of nanoparticle preparation using an oil-in-water emulsification evaporation method.
