# Supplementary material for: Immune Modifying Effect of Drug Free Biodegradable Nanoparticles on Disease Course of Experimental Autoimmune Neuritis
Source: Pharmaceutics. 2022 Nov 8;14(11):2410. doi: 10.3390/pharmaceutics14112410 (PMC9695102; doi:10.3390/pharmaceutics14112410)
Supplement: Supplementary file 1 [file pharmaceutics-14-02410-s001.zip › pharmaceutics-1968157-supplementary.pdf]

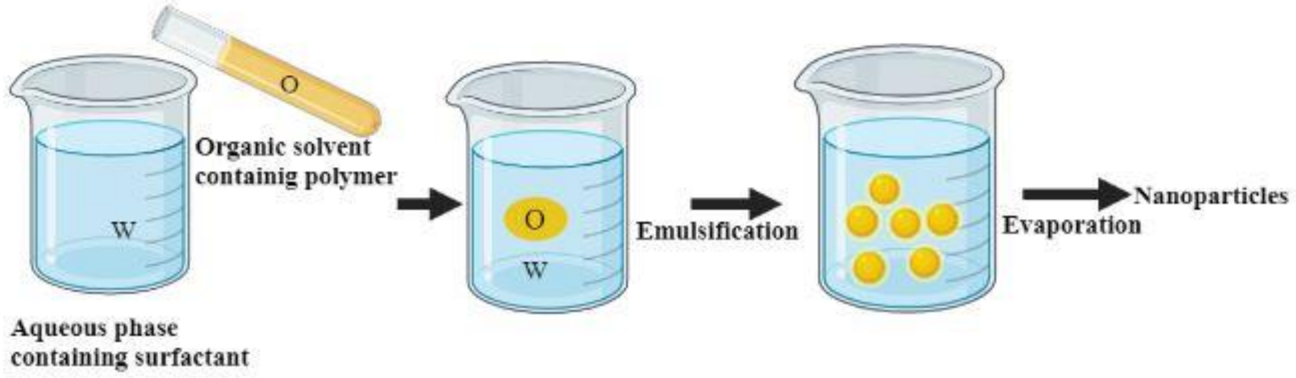

Figure S1. Schematic presentation of nanoparticle preparation using an oil-in-water emulsification evaporation method.
